# Iridociliary melanoma – Clinical case

**Published:** 2016

**Authors:** S Schmitzer, C Butea-Simionescu, A Gheorghe, M Zemba, M Cioboata

**Affiliations:** * Emergency Eye Hospital, Bucharest, Romania; ** OFTALMESTET Clinic, Bucharest, Romania; *** “Dr. Carol Davila” Central Military University Emergency Hospital, Bucharest, Romania

**Keywords:** melanoma, iridocyclectomy, surgery

## Abstract

Abstract

Iris and ciliary body melanoma is an aggressive tumor, which, unfortunately, presents symptoms only in advanced stages and is often discovered accidentally during a routine eye examination. There are several treatment options, ranging from in time monitorization in order to observe the tumor’s evolution to more aggressive methods such as radiotherapy and enucleation.

We present a case of iridociliary melanoma, who underwent conservative surgery, iridocyclectomy under scleral flap, with good results, and maintenance of the function and integrity of the eyeball.

## Introduction

Melanoma of the uveal tract is a cancer that arises from neuroectodermal melanocytes of the iris, ciliary body, or choroid. It is the most common intraocular malignant tumor in Caucasians, with a high potential for hematogenous dissemination [**1**]. The purpose of this article was to discuss the iris and ciliary body melanoma and present a surgical method to solve these cases.

Most patients are asymptomatic and the iridociliary melanoma is detected during a routine ophthalmologic examination. Clinical findings include a hyperpigmented heterogeneous area on the iris, with dilated episcleral sentinel blood vessels. These tumors do not cause pain or other symptoms than in advanced stages with secondary glaucoma or spontaneous tumor necrosis [**1**,**2**].

The diagnosis of iridociliary-pigmented tumor is primarily clinical. Malignancy is suggested by the increase in tumor size, vascularization, and the effect on adjacent ocular tissues [**2**,**3**].

A firm diagnosis requires a more or less specific series of laboratory investigations. In practice, only histopathology offers a positive diagnosis. Unfortunately, tumor biopsy shows the risk of dissemination; however, some authors recommend it for borderline cases clinically benign small tumors [**1**,**3**]. Ultrasound biomicroscopy (UBM) allows the assessment of the tumor structure, its extension to the ciliary body, differentiates between solid or cystic lesions, measures the tumor size [**4**].

The systemic evaluation in suspected iridociliary melanoma include blood count, liver enzymes, chest X-ray, abdominal ultrasound (liver is the main location for metastases), MRI. In some centers, PET-CT is recommended to identify whether there are active lesions. However, 98% of the patients with iridociliary melanoma show no secondary lesions at diagnosis. Those who are found to have metastatic lesions usually have large intraocular tumors with extension to the sclera [**1**,**3**].

If there is uncertainty about the diagnosis and tracking tumor progression is decided, anterior segment photography is absolutely necessary, being at the same time a forensic document.

In terms of histopathology, melanoma may present spindle cells, epithelioid cells or a combination of both. Iris melanoma usually has spindle cells with a better vital prognosis. On the other hand, ciliary body tumors are frequently composed of epithelioid cells with severe long-term prognosis [**5**].

## Aim

The aim of the article was to present a surgical solution for pigmented iris and ciliary body tumors. 

## Case presentation

**Fig. 1:**
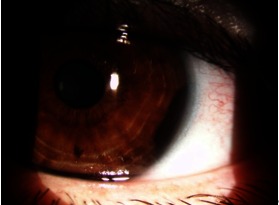
**Fig. 1**Photo of anterior pole before surgery

A 48-year-old female patient presented to our clinic complaining of bilateral progressive decrease in visual acuity without other associated symptoms. The visual acuity exam revealed Right Eye = 0.4 and 0.8 bcva, Left Eye = 4/ 50 bcva, both eyes IOP = 12mmHg and refraction showed Both Eyes: Hypermetropia and Left Eye: Amblyopia. The slit lamp examination of the anterior pole, showed Left Eye: pigmented iris tumor between the hours 3 to 5, extending in the iridocorneal angle with a modified iris stroma (**Fig. 1**). The examination of the eye fundus did not reveal any changes. 

**Fig. 2:**
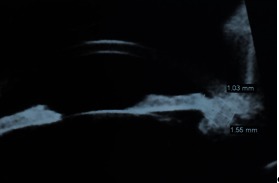
**Fig. 2**Ultrasound biomicroscopy

The ultrasound biomicroscopy showed an iris and ciliary body tumor between the hours 3 to 5, that closed the iridocorneal angle, of approximately 1.03/ 1.55 mm (**Fig. 2**).

**Fig. 3:**
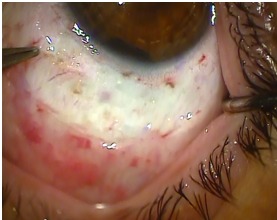
**Fig. 3**After conjunctiva removal

Based on the clinical examination and investigations, a pigmented iridociliary tumor in the left eye was diagnosed and the performance of a conservative surgical intervention like iridocyclectomy under sclera flap was decided. This type of intervention is recommended for small tumors, relatively well defined and apparently compact and consists of the excision of a portion of the iris and the ciliary body with 2mm of healthy tissue around the tumor. The surgery is performed under general anesthesia to avoid any pressure on the globe after retro bulbar anesthesia. It was important to mark the position and size of the tumor excision on the sclera. The conjunctiva was lifted off the area previously marked (**Fig. 3**). 

**Fig. 4:**
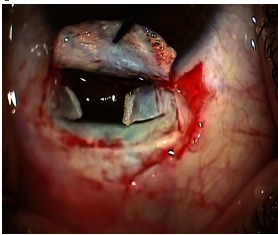
**Fig. 4**H shaped scleral flap with the tumor protruding

A scleral flap of 2/ 3 of the scleral thickness and an exceeding of the tumor with 3mm was performed. Subsequently, the remaining sclera was cut in the shape of H to expose the tumor (**Fig. 4**).

**Fig. 5:**
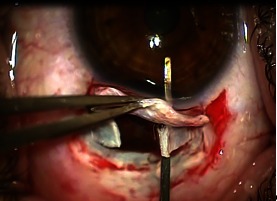
**Fig. 5**Separating the tumor from the sclera

**Fig. 6:**
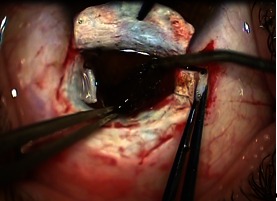
**Fig. 6**Tumor electroexcision

The sclera was lifted from the surface of the tumor with a thin spatula and the tumor excision was performed by using an electrocautery (**Fig. 5-6**).

**Fig. 7:**
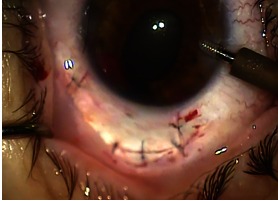
**Fig. 7**After suture of the scleral flap

At the end of the resection, the vitreous must remain intact. If there is a tendency to protrude through the incision, it can be sectioned or anterior vitrectomy can be performed. The two scleral flaps were sutured with 10/ 0 wires. The conjunctiva was sutured in turn with a 10/ 0 wire (**Fig. 7**). 

**Fig. 8:**
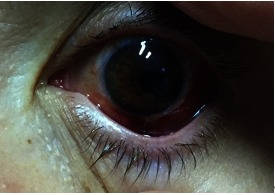
**Fig. 8**Next day after surgery

Postoperatively, the patient presented iris coloboma. The visual acuity was preserved but remained low due to amblyopia (**Fig. 8**). 

This type of surgery presents potential complications after the surgery itself. In time, the iris coloboma may lead to photophobia and unpleasant aspect but can be corrected by pupilloplasty or colored contact lens. Because of the ciliary body excision, there is a high risk of hypotonia; that is why the intervention is contraindicated in cases where the tumor exceeds 3-hour dials or more than 40% of the ciliary body needs to be removed. Also, another complication can be the lens subluxation or its opacity [**6**]. Postoperatively, patients should be checked at every 6 months for 2-3 years and then annually [**1**].

In our case, the histopathology revealed type A spindle cell melanoma cell and the patient was referred to Oncology for a periodic evaluation and complementary treatment.

## Discussion

Factors that influenced the therapeutic decision included tumor size, its location, extrascleral extension, presence of metastases, visual status, age, and general condition of the patient. Tumor size was actually the most important prognostic factor because it was considered that each millimeter of tumor increased the raises in the risk of metastatic tumor growth by 5% [**2**].

The treatment options for iris and ciliary body tumors include the monitoring of the tumor, local radiotherapy, tumor excision by iridocyclectomy and enucleation but each has its own drawbacks [**1**,**3**]. For instance, extensive monitoring of the tumor through a periodic reevaluation is not a long-term option because of the aggressive character of melanoma and sometimes lack of patient compliance. 

Local radiotherapy is a treatment method that has gained much popularity in recent years, despite its side effects, but unfortunately, the costs are high. Plaque radiotherapy consists of a radioactive device sutured to the sclera overlying the tumor. It uses ruthenium-106 and iodine-125 and plates must be at least 3 mm larger than the diameter of the tumor. Local irradiation of large tumors is not recommended because of the risk of scleral necrosis due to the high radiation dose required. Although local radiotherapy induces tumor regression, 10-15% of patients have local recurrences. Also, local radiotherapy leads to a decreased visual acuity by dry eye and severe ocular pain, ischemia, neovascular glaucoma. If these complications occur, enucleation is recommended [**7**]. 

Enucleation is the oldest method of treatment for this type of tumor. Since no data is available that enucleation prolongs survival, it is only recommended in patients with large poorly defined tumors that cause secondary glaucoma, if there is extrascleral extension or if other treatment methods have failed. Statistics showed that approximately 50% of the patients who underwent enucleation, developed metastases later in life because hematogenous dissemination cannot be controlled and microlesions probably existing at the time of enucleation cannot be identified [**8**].

## Conclusions

In our experience, iridocyclectomy is a therapeutic approach with good results, allowing a positive diagnosis through histopathological examination, maintaining visual acuity and keeping the eye over a long period of time; the association of this type of surgery with specific oncological treatment offers a good vital prognosis, with a high survival rate.
